# Alcohol use disorder as a warning sign for depressive disorders in acute psychiatric care? hospitalization demographics in arad (ROMANIA)

**DOI:** 10.1192/j.eurpsy.2021.903

**Published:** 2021-08-13

**Authors:** A. László, M. Mariș

**Affiliations:** 1 Psychiatry Ward, Arad County Emergency Clinical Hospital, Arad, Romania; 2 Psychiatry, Vasile Goldiş Wester University of Arad, Arad, Romania

**Keywords:** alcohol use disorder, Depression, comorbidity, sex differences

## Abstract

**Introduction:**

Screening for psychiatric disorders is the job of primary care providers. As such, general practice residents spend one month on psychiatry rotation. During which, they need to familiarize with the diagnostic and treatment of mental disorders. Since depressive disorders in early stages can be easily overlooked by the untrained eye, we set out to analyse the demographic particularities of our patients.

**Objectives:**

The objective of our study was to analyze demographic characteristics of patients hospitalised on Acute Inpatient Psychiatry Ward with unipolar depression and to identify the specific warning signs, later to be used for an awareness campaign addressed to family medicine residents.

**Methods:**

Data was collected from Acute Inpatient Psychiatry Ward of Arad County Emergency Clinical Hospital (Romania) between 1th January 2019 and 30th September 2020. We included every patient who was discharged with unipolar depression diagnosis according to ICD-10 criterias(F32-F33). Every patient was included only once. In cases of multiple hospitalisations, we included the most severe episode. If the severity of episodes was similar, the longest hospitalisation from the selected period. The data analysis accomplished in Microsoft Excel2010.

**Results:**

A number of 344 patients were included in our analysis (175 male, 169 female). Their presumptive diagnosis upon hospitalisation: Depressive Episode(F32)-32.3%, Recurring Depression(F33)-32,3%, Alcohol Use Disorder(F10)-23,8%, Suicid Attempt(X61-80)-7,0%, Other Psychiatric Disorders(F06,F20-23), Other Substance Use Disorder(F19)-0,6%. From 82 patients with presumptive diagnosis of Alcohol Use Disorder and definitive diagnosis of Depressive Disorder, 90,2% were male.

**Conclusions:**

In primary practice alcohol misuse can be objectively spotted. Awareness is needed to investigate a possibly underlying depressive disorder.

